# Steatohepatitis Is Not Associated with an Increased Risk for Fibrosis Progression in Nonalcoholic Fatty Liver Disease

**DOI:** 10.1155/2018/1942648

**Published:** 2018-07-02

**Authors:** Hannes Hagström, Olof Elfwén, Rolf Hultcrantz, Per Stål

**Affiliations:** ^1^Department of Upper GI, Unit of Liver Diseases, Karolinska University Hospital, Stockholm, Sweden; ^2^Department of Medicine, Huddinge Karolinska Institutet, Stockholm, Sweden

## Abstract

**Introduction:**

Nonalcoholic fatty liver disease (NAFLD) is the most common liver disease globally. The majority of NAFLD patients have fatty liver without inflammation (nonalcoholic fatty liver, NAFL), whereas a minority develop steatohepatitis (nonalcoholic steatohepatitis, NASH). Only NASH and not NAFL has been considered to increase the risk for fibrosis progression. The present study investigates risk factors for fibrosis progression in patients with NAFLD, and if fibrosis progression associates with subsequent mortality.

**Material and Methods:**

All patients with at least two liver biopsies more than a year apart at our hospital between 1971 and 2016 were identified. Data on plausible risk factors for fibrosis progression were collected. Biopsies were scored for the presence of NASH and fibrosis stage. Regression models were used to investigate the association between baseline NASH and fibrosis progression and fibrosis progression with future mortality.

**Results:**

60 patients had undergone serial biopsies (median interval between biopsies 8.4 years, range 1–33 years), with 26 patients (43%) having fibrosis progression. We found no significant risk factors for progression of fibrosis except time between biopsies. Among patients with fibrosis progression, 54% had NAFL and 46% had NASH at baseline. There was a trend for an association between fibrosis progression per se and increased mortality (hazard ratio 2.83, 95% CI 1.0–8.1, *p* = 0.05).

**Conclusions:**

In this study on NAFLD, baseline steatohepatitis was not associated with an increased risk for fibrosis progression. NAFLD patients without steatohepatitis may develop progressive fibrosis, and those with progressive fibrosis appear to have a higher mortality risk irrespective of baseline NASH status.

## 1. Introduction

Nonalcoholic fatty liver disease (NAFLD) is the most common liver disease globally [1] and is projected to become the main indication for liver transplantation in the US in the coming decade [2] and also an increasing indication for liver transplantation in the Nordic countries [3]. Histologically, NAFLD is divided into two major subgroups, nonalcoholic fatty liver (NAFL) and nonalcoholic steatohepatitis (NASH). Approximately 10–20% of patients with NAFLD have NASH, whereas the majority have NAFL [4]. Some patients with NAFLD develop progressive fibrosis, which eventually may progress to cirrhosis. Fibrosis stage correlates well with clinical outcomes and is the strongest predictor for overall and liver-related mortality [5–7]. NAFL has generally been considered a benign condition, and it has been thought that only patients with NASH have a potential for fibrosis progression and cirrhosis development. However, this dogma has recently been challenged in several studies showing that fibrosis progression indeed can occur also in patients with NAFL [8, 9].

However, to date, no study has investigated the impact if fibrosis progression per se is associated with an increased risk of future mortality. We aimed to confirm recent findings from other studies, evaluate risk factors for fibrosis progression, and examine if fibrosis progression per se is associated with increased mortality in NAFLD.

## 2. Materials and Methods

We retrospectively included all patients with NAFLD who had undergone two or more liver biopsies at the Karolinska University Hospital, Stockholm, Sweden in a cohort study. The methodology for the generation of the cohort and ascertainment of NAFLD has previously been described [5, 10]. Briefly, all liver biopsies performed between 1971 and 2009 with a histopathological finding of steatosis were identified, and the corresponding patient charts were examined to ascertain the NAFLD diagnosis. Furthermore, we used a local database created for financial registration based on ICD 10-codes, to identify additional cases that had undergone liver biopsy between 2009 and 2016. All charts were scrutinized from the earliest record, through the time of biopsies and to the death of the patient or to the end of the follow-up period (April 1st, 2016) to exclude liver diseases other than NAFLD. In total, 2176 patients were found to have evidence of fatty liver, with 510 cases being defined as NAFLD. The reason for the baseline liver biopsy was primarily persistent increased serum levels of ALT/AST or the finding of steatosis on a radiological exam. We excluded patients with a follow-up biopsy less than one year after the baseline biopsy or with insufficient clinical data. Likewise, we excluded all patients with any concurrent liver disease or evidence of excessive alcohol consumption, defined as more than 30 grams of alcohol per day in men and more than 20 grams in women.

The cohort was stratified on progression or no progression of fibrosis between the two biopsies. Progression of fibrosis was defined as an increase of at least one stage of fibrosis. In patients who underwent more than two biopsies (*n* = 19), the first and last biopsies were used.

### 2.1. Variables

Data from the time of the baseline and follow-up biopsies were collected from patient charts as per Table 1. Body mass index (BMI) was calculated as weight (kg) divided by height (m) squared. Type 2 diabetes mellitus (T2DM) and hypertension were defined as present if these diagnoses were stated in patient charts or if the patient was using antidiabetic or antihypertensive drugs. After the last biopsy, patients were followed until death of any cause occurred or until the end of the follow-up period (April 1st, 2016). We could ascertain mortality in all 60 patients.

### 2.2. Histological Assessment

Of the baseline biopsies, 54 were available, and for the follow-up biopsies, 41 were available for reevaluation including scoring of necroinflammatory changes and NASH. Available liver biopsies were reevaluated by two of the researchers (RH and HH), blinded to patient characteristics. The stage of fibrosis and the NAFLD activity score were determined according to Kleiner et al. [11]. The FLIP algorithm was used to define the presence of NASH [12]. For cases where the archival biopsy could not be retrieved, scoring of the fibrosis stage was obtained from the original pathology report, since the interagreement assessment regarding the fibrosis stage between the original report and the follow-up review was high (kappa 0.80). However, in these cases the original scoring of steatosis, lobular inflammation, and ballooning were not included in the analysis of data.

### 2.3. Statistical Analysis

Descriptive statistics for continuous variables are expressed as median (range), and categorical variables are presented as absolute numbers (percentages). Differences in continuous variables were analyzed using the Mann–Whitney *U* test and differences in categorical variables with Fisher's exact test. The median rate of fibrosis progression in patients with baseline NASH and NAFL, respectively, was calculated as the increase in fibrosis stages divided by time between biopsies, measured in years. A logistic regression model was used to identify factors at baseline and follow-up associated with progression of fibrosis. In multivariate modeling, we adjusted for a number of a priori defined possible confounders including sex, age, BMI, T2DM at baseline, and time between biopsies. A Cox regression model was used to investigate histological factors at baseline as well as fibrosis progression per se and their association with mortality after the second liver biopsy. These analyses were adjusted for the same confounders as in the logistic regression model.

As NASH might be more important in early stages of fibrosis, we performed sensitivity analyses excluding cases with advanced fibrosis (stages 3-4). All analyses were performed using STATA v 13.0 (StataCorp, College Station, Texas, USA). A two-tailed *p* value of <0.05 was considered as statistically significant.

### 2.4. Ethical Considerations

The study was conducted in accordance with the Helsinki Declaration of 1975, as revised in 1983, and approved by the Ethics Committee at Karolinska University Hospital, Stockholm, Sweden (Dnr: 2011/905-31/2 and 2015/1591-32).

## 3. Results

### 3.1. Study Population

Among the 510 patients with a histological diagnosis of NAFLD at baseline, 82 underwent a second biopsy. Of these, 17 patients were excluded due to missing clinical data from the time of the follow-up biopsy, and five patients were excluded because the second liver biopsy was performed within one year of the first, leaving 60 patients for analysis.

**Figure 1:**
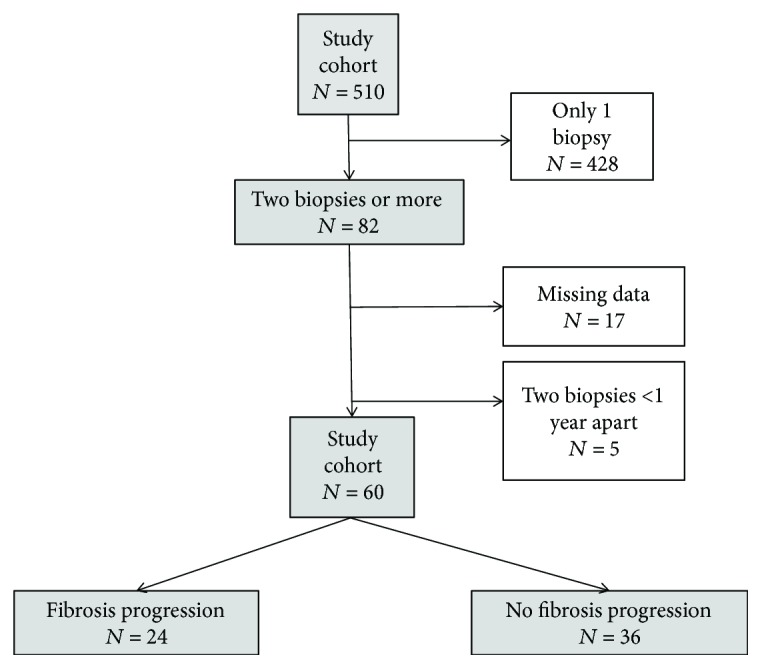
Flowchart for patient inclusion.

A flowchart for patient inclusion is presented in Figure 1. The main reasons for performing the follow-up biopsy were clinical follow-up of steatosis diagnosed at the first biopsy (*n* = 28), persistent high levels of ALT/AST (*n* = 15), while other causes included suspected cirrhosis on ultrasound, staging of fibrosis, or ruling out other suspected diseases (*n* = 17). The cohort was followed from the index biopsy to the death of the patient or until the end of the study period for a median time of 24.8 years (range 7.5–41.2). The median total follow-up time was similar in patients with (24.8 years) and without (25.3 years) fibrosis progression (*p* = 0.85).

### 3.2. Cohort Characteristics

Clinical, histological, and laboratory features of the cohort are summarized in Table 1. The median age of the cohort at baseline was 46 years (range 19–70 years). There were 62% male participants, and 17% had T2DM. Among the 54 patients with baseline biopsies available for reevaluation, 48% (*n* = 26) had NAS between 1 and 4 and 52% (*n* = 28) had NAS between 5 and 8. The median baseline NAS score was 5 (range 1–8), and NASH was present in 33 (61%) of patients.

Fibrosis staging at the baseline biopsy disclosed stage 0 in 15 (25.0%), stage 1 in 22 (36.7%), stage 2 in 14 (23.3%), stage 3 in 6 (10.0%), and stage 4 in 3 (5.0%) patients.

### 3.3. Histological Evolution of NAFLD during Follow-Up

The median follow-up interval between the first and second liver biopsies was 8.4 years (range 1–34 years). Twenty-seven patients (45%) had liver biopsies more than ten years apart. A total of 26 (43%) patients had fibrosis progression while 34 (57%) patients had stable or regression of fibrosis. Time between the index and the follow-up biopsy was significantly longer in the group with fibrosis progression (median 16.2 versus 5.5 years, *p* = 0.01). Differences at baseline and at follow-up between patients with and without fibrosis progression are presented in Table 2.

The distribution of fibrosis stages at the baseline and follow-up biopsies is shown in Table 3. Thirteen patients progressed by one stage, ten patients by two stages, two patients by three stages, and one patient progressed by four stages. At follow-up liver biopsy, 21 patients had advanced fibrosis of which 9 had stage 3 and 12 had stage 4 (cirrhosis).

Among the 41 patients with both baseline and follow-up biopsies available for scoring of steatosis, lobular inflammation and ballooning, 23 (62.1%) had NASH at baseline and 18 (48.7%) at follow-up. Of the 23 patients with baseline NASH, 10 (43.5%) experienced resolution of NASH, while 13 (56.5%) had NASH also at follow-up. Of the 14 patients without baseline NASH, five (35.7%) developed NASH while 9 (64.3%) did not.

The median rate of fibrosis progression was 0.15 stages/year (range 0.03–0.78) for patients with NASH at baseline and 0.11 stages/year (range 0.03–0.59) for patients with baseline NAFL (*p* = 0.91).

The presence of NASH at baseline was not associated with fibrosis progression in the multivariate logistic regression analysis (aOR 1.04, 95% CI 0.30–3.64, *p* = 0.96) (Table 4(a)). We found no parameter at baseline or follow-up that predicted fibrosis progression including NAS, baseline fibrosis stage, age, sex, BMI, T2DM, hypertension, or biochemical parameters (data not shown). As predicted, time between biopsies was significantly associated with fibrosis progression (aOR 1.08 for each year between biopsies, 95% CI 1.02–1.14, *p* = 0.01). Further adjusting the model for time between biopsies did not affect the estimates significantly (aOR for NASH at baseline 1.02, 95% CI 0.97–1.07, *p* = 0.46). Importantly, both patients with and without fibrosis progression had a similar development of BMI and type 2 diabetes during follow-up (Table 2). In patients with fibrosis progression, 9/26 (35%) had a reduction in BMI between the two biopsies while none had resolution of type 2 diabetes.

### 3.4. Differences in Fibrosis Progression between NAFL and NASH

Among the 26 patients with fibrosis progression on the follow-up biopsy, 24 had biopsies available for baseline biopsy scoring. Of these, 10 (42%) had NAFL and 14 (58%) had NASH at the baseline biopsy. There were no significant differences at baseline concerning steatosis, lobular inflammation, ballooning, NAS, or fibrosis stage between patients with and without fibrosis progression (Table 5).

### 3.5. Mortality during Follow-Up

After the follow-up biopsy, patients were followed for a median of 6.7 years (range 0.1–34.0). During this time, 21 patients (35%) died, eight (25%) in the group without fibrosis progression and thirteen (50%) in the group with fibrosis progression (*p* = 0.05).

In the Cox regression model, only fibrosis progression was borderline associated with mortality (aHR 2.83, 95% CI 1.00–8.05, *p* = 0.051), while neither presence of NASH nor a high NAS (5–8) at baseline or follow-up showed any trend to an association with increased mortality (Table 4(b)).

### 3.6. Sensitivity Analysis

No association between baseline NASH and fibrosis progression or mortality was found when excluding cases with fibrosis stages 3-4 from the analysis (data not shown).

## 4. Discussion

In this retrospective cohort study of 60 NAFLD patients with sequential liver biopsies, a similar proportion of patients with NAFL and NASH had evidence of fibrosis progression. Thus, we confirm similar findings from other groups, which together strengthen the hypothesis that fibrosis progression also can occur in NAFLD patients without steatohepatitis at an initial baseline liver biopsy.

In addition, we found a trend towards increased mortality in patients with fibrosis progression, irrespective if NASH was present or not in the baseline liver biopsy and after adjustment for confounders. This finding is in line with results from previous studies implying that the fibrosis stage correlates with clinical outcomes and is the strongest predictor for overall and liver-related mortality [5–7]. However, due to the retrospective nature of this study, a risk of selection bias may be present. Cases with baseline NAFL and progressive fibrosis could have been selected for follow-up due to clinical events and suspicion of fibrosis progression. Nevertheless, it is obvious that some cases with NAFL without steatohepatitis have a risk for fibrosis progression, and future studies are needed to identify risk factors for fibrosis progression in larger cohorts of patients without NASH. Taken our data together with those from other groups [8, 13–15], it is clear that progression of liver fibrosis progress can occur in both NAFL and NASH.

The only significant risk factor for progression of fibrosis in the present study was the time between the two biopsies. In contrast, baseline T2DM and BMI were also associated with fibrosis progression in three recent studies with similar design as ours [8, 14, 15]. These studies differed from ours by having a greater proportion of patients with T2DM and also with a higher BMI at baseline. McPherson et al. included 108 patients who underwent two liver biopsies at least one year apart with a median follow-up interval of 6.6 years. In that cohort, T2DM was an independent predictor of fibrosis progression [8]. Forty-two percent of their patients had fibrosis progression, and 43% had advanced fibrosis in the follow-up biopsy. Our cohort had a longer follow-up interval (8.4 years), and 35% had advanced fibrosis on the follow-up biopsy. Since patients in our cohort had lower BMI (26.4 kg/m^2^) and a lower prevalence of T2DM (17%), our cohort may possibly be more comparable to the general population. The prevalence of diabetes was 5% in the general Swedish population in 2012 [16], and the prevalence of obesity was 10% in a study from 2006 [17]. Nonetheless, the smaller fraction of patients with T2DM and a relatively low median BMI in our cohort limit our ability to assess the impact of diabetes and obesity on fibrosis progression. Therefore, we cannot exclude the possibility of a type 2 error in this respect.

### 4.1. Strengths and Limitations

The major strength of this study was the long follow-up duration. The follow-up interval between the first and second liver biopsies is the longest hitherto documented. A long enough follow-up time is essential when studying fibrosis progression in patients with NAFLD, since this is a slow process. In a recent meta-analysis, the rate of fibrosis progression was estimated to one stage per seven years in subjects with NASH and per 14 years in those with NAFL [13]. Furthermore, the access to highly valid outcome data on mortality after the second biopsy, with no missing data, is another strength. Also, the cohort was more comparable to the general population than those of previous studies regarding BMI and the presence of type 2 diabetes. The main indication for the first biopsy was elevated liver transaminases and not suspicion of cirrhosis, reflected by the low percentage of patients (15%) having advanced fibrosis at baseline, which suggests a low selection bias at the first biopsy in our study.

The primary limitation of this study is a possible selection bias at the second biopsy, since cases with progressive fibrosis are probably more likely to undergo repeat examination than cases without. However, liver biopsies were historically performed more frequently compared to today, due to a lack of other modalities. Eighty-three percent of patients in this study were originally investigated before year 2000, thus reducing (but not excluding) the risk of selection bias. Since the biopsies were not planned per a specific protocol, the follow-up interval varies to a wide extent. Longer follow-up intervals may have an overrepresentation of older patients with more severe liver disease. As seen in Table 3, the proportion of patients with advanced fibrosis (stages 3 and 4) more than doubled from the index to the follow-up biopsy. In the analysis, we therefore accounted for this by adjusting the statistical models for time between biopsies.

Other limitations include the small sample size, the lack of data on NASH at follow-up biopsy in 19 cases, and the absence of genetic status including *PNPLA3* polymorphisms due to lack of stored blood samples.

A major limitation of all paired biopsy studies in NAFLD, including ours, is the risk of residual confounding. Such confounding factors could comprise of lifestyle changes, including a change in alcohol consumption or medications during follow-up.

We did have access to data on development of BMI and type 2 diabetes, which were not associated with progression of fibrosis. Indeed, 35% of patients with fibrosis progression had a reduction in BMI during the follow-up period, suggesting that a reduction of BMI per se might not be enough to reduce the risk of fibrosis progression in some patients.

### 4.2. Implications for Clinical Practice and Future Research

The clinical impact of this study together with the conclusions from previous studies [8, 14, 15] is that progressive fibrosis can occur in both NAFL and NASH. This indicates that patients with steatosis but no inflammation on index liver biopsy should not be routinely excluded from clinical follow-up. The results do not help us to identify significant predictors of fibrosis progression, due to the limited size of the cohort, which thus must be a focus of future studies.

The rate of fibrosis progression was quite slow in this study but comparable to what has been found in a recent meta-analysis [13], indicating that repeat noninvasive measurements of fibrosis, such as transient elastography, could be performed with relatively long intervals.

Our study also demonstrates a trend towards higher mortality in patients with progressive fibrosis, which may have implications for selecting patients intended for more intense follow-up. This finding has to be confirmed in future studies on larger cohorts with a long enough follow-up time, which also should aim to find predictors for fibrosis progression. The present study could possibly contribute to future meta-analyses on this topic.

## 5. Conclusions

Both NAFL and NASH can lead to progressive fibrosis, with a mean fibrosis progression rate in the present study from 0.11 to 0.15 stages per year. Baseline steatohepatitis was not associated with an increased risk for fibrosis progression. Patients with progressive fibrosis appear to have a higher risk for mortality, regardless if NASH is present or not at baseline. Larger studies are needed to identify which patients with NAFL are at an increased risk for fibrosis progression.

## Figures and Tables

**Table 1:** Clinical characteristics at baseline.

Parameter	*N* (%) or median	Range
Age (years)	46	19–70
Sex, male, *N* (%)	37 (62)	
BMI (kg/m^2^)	26.4	21.2–38.7
Smoking, ever, *N* (%)	21 (37)	
T2DM, *N* (%)	10 (17)	
Hypertension, *N* (%)	14 (23)	
Platelets (×10̄^9^/L)	256	56–347
ALT (IU/L)	88	10–307
AST (IU/L)	46	14–289
Ferritin (*μ*g/L)	133	44–753
ALP (IU/L)	200	47–876
GGT (IU/L)	78	22–599
Bilirubin (mg/dL)	0.58	0.18–2.22
PK-INR	1	0.9–1.4
Albumin (g/dL)	4.4	3.2–5.3
AST/ALT ratio	0.7	0.4–4.5
CRP (mg/L)	10	1–26
Cholesterol (mg/dL)	221	77–402
Triglycerides (mg/dL)	186	44–460
Glucose (mg/dL)	95	67–305
NAS (0–8)	4	1–8
NASH *N* (%)	33 (61)	
Fibrosis stage		
0 *N* (%)	15 (25)	
1 *N* (%)	22 (37)	
2 *N* (%)	14 (23)	
3 *N* (%)	6 (10)	
4 *N* (%)	3 (5)	

Abbreviations: BMI: body mass index; DM2: diabetes mellitus type 2; ALT: alanine aminotransferase; AST: aspartate aminotransferase; ALP: alkaline phosphatase; GT: gamma glutamyltransferase; NAS: nonalcoholic fatty liver disease activity score.

**Table 2:** Comparison of clinical characteristics at baseline and follow-up between patients with and without progression of fibrosis.

	No fibrosis progression *N* (%) or median (*N* = 34)	Range	Fibrosis progression *N* (%) or median (*N* = 26)	Range	*p* value
*Baseline biopsy*					
Sex, *N* male (%)	22 (65)		15 (58)		0.6
Age (years)	42.5	19–70	50	20–66	0.28
BMI (kg/m^2^)	26.7	23.5–38.7	26.6	21.2–32.2	0.29
T2DM, *N* (%)	6 (18)		4 (15)		0.58
Hypertension, *N* (%)	7 (21)		7 (27)		0.4
Platelets (×10^9^/L)	265	113–347	233	56–332	0.48
ALT (U/L)	92	22–307	86	10–264	0.53
AST (U/L)	49	29–135	46	14–288	0.64
Bilirubin (mg/dL)	0.58	0.35–2.22	0.53	0.16–1.29	0.65
Albumin (g/dL)	4.4	3.3–5.3	4.4	3.2–5.2	0.84
PK-INR	1.0	0.9–1.3	1.0	1.0–1.2	0.45
Total cholesterol (mg/dL)	232	77–402	189	127–340	0.09
NASH, yes (%)	19/30 (63)		14/24 (58)		0.71
*Follow-up biopsy*					
Years of follow-up	5.5	1.1–27.2	16.2	1.7–33.7	0.01
Age (years)	52	28–83	66	21–84	<0.01
BMI (kg/m^2^)	27.8	24.7–38.7	27.1	21–35	0.3
Delta BMI	1.9	−7.5 to 6.4	0.8	−5.0 to 8.1	0.29
T2DM, *N* (%)	18 (53)		12 (46)		0.4
Hypertension, *N* (%)	13 (38)		11 (42)		0.48
Platelets (×10^9^/L)	238	61–465	215	92–505	0.25
ALT (U/l)	59	11–343	64	17–604	0.62
AST (U/I)	41	13–238	53	19–299	0.04
Bilirubin (mg/dL)	0.58	0.23–1.87	0.79	0.23–5.73	0.22
Albumin (g/dL)	4.2	1.7–5.2	3.7	2.0–4.8	0.007
PK-INR	1.0	0.9–1.3	1.0	0.9–1.6	0.01
Total cholesterol (mg/dL)	208	93–347	178	131–290	0.17
NASH, yes (%)	10/24 (42)		9/17 (53)		0.54

Abbreviations: BMI: body mass index; DM2: diabetes mellitus type 2; ALT: alanine aminotransferase; AST: aspartate aminotransferase; ALP: alkaline phosphatase; GT: gamma glutamyltransferase; NAS: nonalcoholic fatty liver disease activity score.

**Table 3:** Distribution of the fibrosis stage at baseline and follow-up liver biopsies.

Baseline fibrosis stage	Follow-up fibrosis stage					
	Stage 0	Stage 1	Stage 2	Stage 3	Stage 4	Total
Stage 0	6	**5**	**2**	**1**	**1**	15
Stage 1	2	13	**3**	**3**	**1**	22
Stage 2	1	3	3	**2**	**5**	14
Stage 3	0	1	0	2	**3**	6
Stage 4	0	0	0	1	2	3
Total	9	22	8	9	12	60

Cases with fibrosis progression are marked in bold.

**Table:** (a) Steatohepatitis at baseline biopsy and association with fibrosis progression.

Parameter	OR	95% CI	*p* value	aOR^1^	95% CI	*p* value
NASH	0.81	0.27–2.43	0.71	0.97	0.25–3.69	0.96
NAS 5–8	0.88	0.30–2.56	0.81	0.74	0.21–2.62	0.65

^1^Adjusted for age at first biopsy, sex, BMI, type 2 diabetes, and time between biopsies. Abbreviations: OR: odds ratio; aOR: adjusted odds ratio; CI: confidence interval; NASH: nonalcoholic steatohepatitis; NAS: NAFLD activity score.

**Table:** (b) The respective association between NASH at baseline, high NAS at baseline, and fibrosis progression per se, with mortality using Cox regression.

Parameter	HR	95% CI	*p* value	aHR^1^	95% CI	*p* value
NASH	1.43	0.55–3.69	0.46	0.97	0.20–4.67	0.97
NAS 5–8	1.02	0.41–2.54	0.97	0.46	0.12–1.69	0.24
Fibrosis progression	2.10	0.87–5.09	0.10	2.83	0.99–8.05	0.051

^1^Adjusted for sex, age, BMI, type 2 diabetes at first biopsy, and time between biopsies. Abbreviations: OR: odds ratio; aOR: adjusted odds ratio; CI: confidence interval; NASH: nonalcoholic steatohepatitis; NAS: NAFLD activity score.

**Table 5:** Histological disease activity at baseline and follow-up in patients with and without fibrosis progression. Fibrosis stage was determined in all biopsies.

Parameter	No fibrosis progression (mean)	SD	Fibrosis progression (mean)	SD	*p* value
*Baseline* ^∗^					
Steatosis (1–3)	1.73	0.94	1.79	0.88	0.88
Lobular inflammation (0–3)	1.43	0.85	1.33	0.7	0.63
Ballooning (0–2)	0.97	0.85	0.88	0.85	0.69
NAS (0–8)	3.86	2.31	3.84	2.17	0.92
Fibrosis (0–4)	1.47	1.16	1.15	1.04	0.28
NASH (number of patients, %)	19/30	63%	14/24	58%	0.78
*Follow-up* ^∗^					
Steatosis (1–3)	1.21	0.93	1.12	0.78	0.83
Lobular inflammation (0–3)	1.17	0.92	1.29	0.69	0.52
Ballooning (0–2)	0.54	0.78	0.94	0.83	0.11
NAS (0–8)	2.92	1.98	3.35	1.73	0.43
Fibrosis (0–4)	1.18	1.11	2.81	1.17	<0.001
NASH (number of patients, %)	10/24	42%	9/17	53%	0.54

^∗^54 biopsies were available for scoring of NASH at baseline and 41 at follow-up. Abbreviations: NAS: NAFLD activity score; NASH: nonalcoholic steatohepatitis; SD: standard deviation.

## Data Availability

The data used to support the findings of this study are available from the corresponding author upon reasonable request.
